# *Moringa oleifera* Supplementation Improves Antioxidant Status and Biochemical Indices by Attenuating Early Pregnancy Stress in Beetal Goats

**DOI:** 10.3389/fnut.2021.700957

**Published:** 2021-07-23

**Authors:** Ali Afzal, Tarique Hussain, Amjad Hameed

**Affiliations:** ^1^Animal Sciences Division, Nuclear Institute for Agriculture and Biology College, Pakistan Institute of Engineering and Applied Sciences (NIAB-C, PIEAS), Faisalabad, Pakistan; ^2^Nuclear Institute for Agriculture and Biology College, Pakistan Institute of Engineering and Applied Sciences (NIAB-C, PIEAS), Faisalabad, Pakistan

**Keywords:** *Moringa oleifera*, pregnancy stress, antioxidants status, beetal goats, biochemical indices of blood plasma

## Abstract

This study investigated the effects of supplementing different levels of *Moringa oleifera* leaf powder (MOLP) on antioxidant status and blood biochemical indices during early gestation in Beetal goats. A total of 30 goats were randomly divided into three groups (*n* = 10) comprising control (basal diet without MOLP), the 1.6% MOLP supplemented group (basal diet + 1.6% MOLP), and the 3.2% MOLP supplemented group (basal diet + 3.2% MOLP). The experiment started 5 days before estrus synchronization and lasted till day 60 of gestation. The MOLP significantly increased plasma flavonoids in 1.6% as well as 3.2% supplemented group on days 40 and 60 of pregnancy, while total phenolic contents were observed to be higher in the 3.2% MOLP supplemented group throughout the experiment in comparison with the control group. The supplementation improved plasma total antioxidant capacity (TAC) by decreasing malondialdehyde (MDA) and total oxidant status (TOS) in contrast to the control group. The activities of superoxide dismutase (SOD) and peroxidase (POD) were enhanced in both supplemented groups, whereas catalase (CAT) activity was noticed to be significantly high in the 3.2% MOLP supplemented group. The protein contents were significantly elevated with 1.6 and 3.2% supplementation levels from day 40 to day 60 of the experiment. Plasma sugar level, carotenoids, progesterone profile, and hydrolytic (protease and amylase) enzymes activities were improved only when supplemented with 3.2% MOLP. The findings suggest that supplementing with 3.2% MOLP provides beneficial effects on early pregnancy stress in Beetal goats.

## Introduction

Pregnancy is a physiological process characterized by an intense metabolic burden that interrupts antioxidant stability and energy balance. It is common for about 25% of the embryos die or be reabsorbed within 2 weeks of pregnancy before implantation (1). The removal of zona pellucida of the embryo is followed by an increase in reactive oxygen species (ROS) production (2). The overloading of ROS promotes the activity of nicotinamide adenine dinucleotide phosphate hydrogen (NADPH) oxidase and suppresses antioxidant enzymes such as glutathione peroxidase (POD), catalase (CAT), and superoxide dismutase (SOD) (3). The disturbance in oxidant and antioxidant balance during early pregnancy may cause severe damage to lipid membranes, proteins, and DNAs, which arrests the further development and growth of the embryo (4).

The early pregnancy nutritional requirements are more than normal maintenance because goats need more energy to combat oxidative stress and maintain a healthy pregnancy (5). However, maternal malnutrition is very common around the world in small ruminants due to the high cost of feed, particularly in developing countries. Therefore, the small ruminants are mainly kept on conventional grazing pastures, low-quality hay, and crop by-products. These feedstuffs lack an adequate amount of protein, vitamins, and minerals to fulfill the nutritional requirements of pregnant goats (6). Maternal malnutrition has a negative impact on the conception rate and also adversely affects the fetus growth (7). Supplementation of the diet with appropriate phytobiotics (plant-derived feed additives) having suitable nutritional values has been proposed as one of the potential approaches for improving the antioxidant and nutritional status of goats during pregnancy (8–10). Phytobiotics from herbal plants are a balanced source of different nutrients that have been explored to have high antioxidant and immunomodulatory properties and may serve as a potential feed supplement for ruminants (11, 12).

*Moringa oleifera* (MO) is an evergreen multipurpose tree of high economic importance and nutritional values (13). Leaves of MO, the most nutritious and utilized part of the plant, are a rich source of proteins, amino acids, minerals, and vitamins (14). Generally, the dried leaves contain 19 different amino acids and an abundance of bioactive compounds, especially antioxidant substances such as flavonoids (myricetin, quercetin, and kaempferol); phenolic acids (gallic, chlorogenic, and ellagic acid); proanthocyanidins; vitamin E; vitamin C; selenium, zinc; and β carotene. These compounds are present separately in different plants but MO is unique in having them all in appreciable amounts, and they have also been reported to possess strong antioxidant potential than synthetic antioxidants such as rutin and butylated hydroxytoluene (15). The utilization of MO as an animal feed supplement in tropical and subtropical zones has several advantages over other plants, such as its potential to tolerate both drought and mild frost conditions, to be a multicut fodder with short cutting interval, to be easily adapted and digested by the animals, and to have high mineral (Ca and Fe) and protein contents, essential fatty acids, and negligible amounts of antinutritional factors (16).

The MO has been reported to improve reproductive performance and serum antioxidant status during pregnancy in mice (17). Previous studies provided information about the impact of MO on lactation performance and milk quality in dairy cows and goats (9, 18). However, there is little information about dietary supplementation impacts of MO on early pregnancy stress in goats. Therefore, the study was designed to investigate the effects of *Moringa oleifera* leaf powder (MOLP) supplementation on plasma antioxidant status and different biochemical indices during early pregnancy in Beetal goats.

## Materials and Methods

### Ethical Statement

The experimental plan and ethical clearance were approved and granted by the Institutional Research Committee for Animal Use and Care at Nuclear Institute for Agriculture and Biology (NIAB), Faisalabad, Pakistan.

### Study Design and Animal Management

The experiment was carried out at the goat farm of the Nuclear Institute for Agriculture and Biology (NIAB) located at about 7 km from the center of Faisalabad city, Pakistan (latitude 31.4287°N and longitude 73.0791°E) with an altitude of 184 m above sea level. The average temperature and rainfall were 24°C and 16.66 mm, respectively, during the months of January to March 2020 of the experiment.

**Figure 1:**
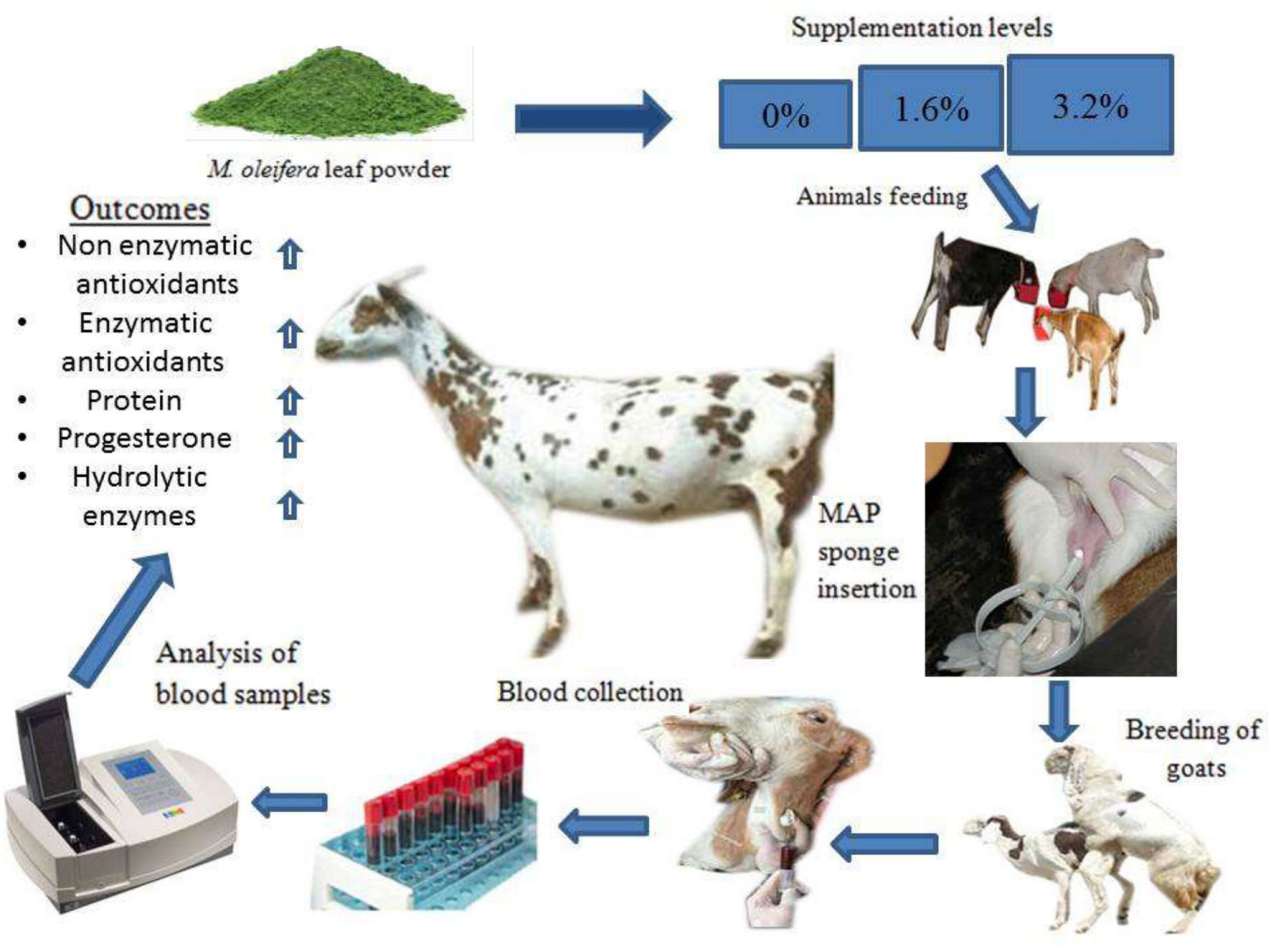
Graphical abstract.

**Figure 2:**
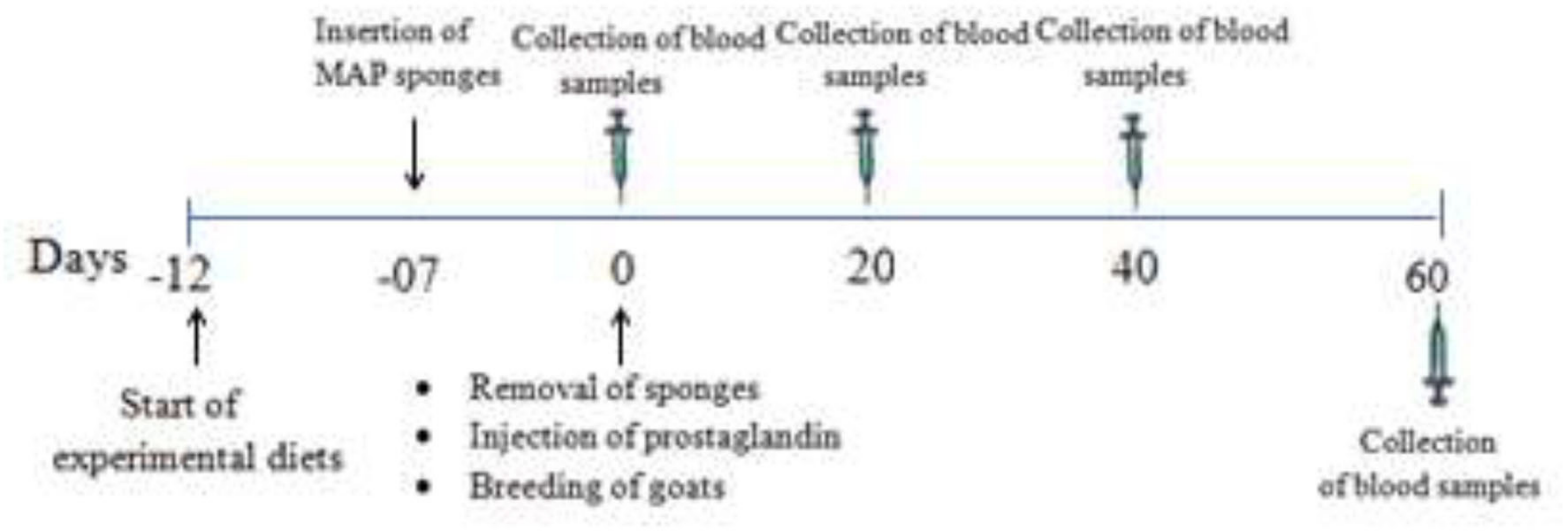
Schematic diagram of experiment protocols.

A total of 30 multiparous, clinically healthy, non-pregnant Beetal goats about 2–3 of years of age, weighing about 40 ± 2.8 kg, and with a body condition score of 3.12 ± 1.20 were selected. The animals were randomly (*n* = *10*) divided into control group (250 g basal diet without MOLP per animal per day), 1.6% MOLP supplemented group (250 g basal diet with 4 g MOLP per animal per day), and 3.2% MOLP supplemented group (250 g basal diet with 8 g MOLP per animal per day). The basal diet consisted of wheat, corn, rice bran, soybean meal, sugarcane molasses, and minerals formulated to fulfill the nutritional requirements of the goats (19) as shown in Table 1. The feeding levels were adjusted according to the previous studies conducted on mice with some modification in the goats (17, 20). The does were offered free pasture (*Chloris gayana, Carduus nutans, Leptochloa fusca, Cirsium arvense*, and *Chenopodium album*) grazing two times during the morning and evening schedule and were provided free access to clean drinking water. The animals were kept in well-ventilated semiopen sheds during the experiment. The experiment started 5 days prior to estrus synchronization and lasted till day 60 of pregnancy. All the animals were provided an adaptation period of 1 week for experimental diets. Before the commencement of the experiment, deworming of the does was done with Albendazole (Zoben, Prix, Lahore, Pakistan) at a dosage of 2.5 mg/5 kg of the body weight. Blood samples (5 ml) were collected from the jugular vein in sterile EDTA tubes (Vacutainer, Xinle) with a 20-day interval except for hormone (progesterone) analysis, which was collected after a 15-day interval from the day of conception (day 0). Plasma separation was done by centrifugation at 3,000 rpm/4°C and stored at −20°C for biochemical analysis. The graphical abstract and schematic diagram of experiment protocols are shown in Figures 1, 2.

**Table 1:** Basal diet formulation (% DM).

**Ingredients**	**Amount (%)**
Wheat grains	30
Wheat bran	15
Wheat straw	10
Corn	20
Rice bran	15
Soyabean meal	5
Molasses	3
Dicalcium phosphate	1.8
Vitamin and mineral premix^a^	0.2
Total	100

a *Vitamin and mineral premix per Kg containing: Vit A 50,000 IU, Vit D3 80,000 IU, Vit E 300 IU, P 3.5%, Ca 18.5%, Mg 8.2%, Na 3%, Cu 800 mg, Zn 3,200 mg, Mn 3,333 mg, Iodate 68 mg, Co 16 mg, Se 24 mg*.

### Estrus Synchronization

Estrus synchronization of the does was done using medroxyprogesterone acetate polyurethane (MAP) sponges. The sponges were made using the previous protocol developed for ewes with some modifications for goats (21, 22). Briefly, polyurethane sponges (length 3 cm and diameter 2 cm) were impregnated with 60 mg medroxyprogesterone acetate (Depo Provera; The Pfizer, Belgium), dissolved in 3 ml ethanol, and then left overnight in the fume hood for drying. The sponges were dusted with an antibiotic powder (streptomycin/penicillin) just before their insertion into the cranial part of the vagina with the help of a speculum and a sterilized applicator lubricated with paraffin oil. The sponges were placed for 7 days, and on the day of removal, all the does were injected with prostaglandin (Cloprostinol; Synchromate^®^ @ 125 μg, Bremer, Germany). After showing behavioral estrus signs, a natural breeding service was provided using fertile bucks. Pregnancy determination was based on progesterone profile and non-return to estrus.

### Source and Processing of Plant Material

The fresh, green MO (PKM_1_) leaves were obtained from a breeder farm (Lahore, Pakistan), and their authenticity was confirmed by a concerned botanist at NIAB. A representative sample was submitted to the herbarium, Department of Botany, University of Agriculture, Faisalabad, Pakistan for future reference. The leaves were washed properly and dried under shade till constant weight. The dried leaves were ground to make a fine powder and stored in air-tight containers for future use.

### Chemical Analysis of Diet

The basal diet and MOLP were analyzed for dry matter, crude protein, crude fat, ash, nitrogen-free extract, neutral detergent fiber, and acid detergent fiber by using the standardized protocols of analytical chemists (23). The chemical composition of the basal diet is shown in Table 2.

**Table 2:** Chemical composition of basal diet and MOLP (Dry matter basis).

**Constituents (%)**	**Basal diet**	**MOLP**
	**Mean ± SE**	**Mean ± SE**
Dry matter	94.8 ± 0.43	92.5 ± 0.52
Crude protein	13.5 ± 0.16	18.2 ± 0.06
Crude fat	4.4 ± 0.23	5.5 ± 0.05
Ash	16.7 ± 0.37	11.3 ± 0.03
Nitrogen free extract	47.3 ± 0.33	38.4 ± 0.29
Neutral detergent fiber	53.2 ± 0.26	32.4 ± 0.12
Acid detergent fiber	18.06 ± 0.13	19.1 ± 0.08

*MOLP, Moringa oleifera leaf powder*.

## Biochemical Analysis of MOLP

The MOLP was analyzed for its various biochemical components as shown in Table 3.

**Table 3:** Biochemical/nutritional constituents of *Moringa oleifera* leaf powder.

**Biochemical constituents**	**Mean ± SE**
Total flavonoids (μg/g)	258.58 ± 2.28
Total phenolic contents (μM/g)	36, 000 ± 3.21
Total sugars (mg/g)	27.51 ± 1.52
Vitamin C (μg/g)	546.16 ± 3.06
Total carotenoids (mg/g)	13.87 ± 0.33
Lycopene (mg/g)	9.95 ± 0.17
Methionine (% of DM)	0.42 ± 0.012
Lysine (% of DM)	1.51 ± 0.057
Calcium (mg/g)	180 ± 1.154
Sodium (mg/g)	2.13 ± 0.075
Potassium (mg/g)	8.99 ± 0.571
Iron (mg/g)	0.16 ± 0.034

### Total Flavonoids

The amount of total flavonoids (TF) was determined as described by Lin and Tang (24). The MOLP methanol extract (2 mL) was homogenized with 0.1 mL of AlCl3 (10%), 2.8 mL of deionized water, and 0.1 mL of potassium acetate (1 M). After an incubation of 40 min at 37°C, the absorbance of the assay mixture was measured at 415 nm by a spectrophotometer (UV-VIS U2800, Hitachi, Japan). Rutin was used as the standard, and the TF content was measured as microgram RE gram^−1^ of the sample.

### Total Phenolic Contents

The total phenolic contents (TPC) were estimated by a micro colorimetric assay as described by Ainsworth and Gillespie (25) with some modifications. The MOLP (0.5 g) was dissolved in 500 μL ice-cold methanol (95%) and incubated at 37°C for 48 h in the dark. Then, its centrifugation was done for 5 min at 14,462 × g. The supernatants were separated and used for the estimation of TPC. For this purpose, 100 μL of supernatant was mixed with 100 μL of F-C reagent 10% (v/v), was vortexed thoroughly, and then was incubated at 37°C for 1 h after adding 800 μL of sodium carbonate (Na_2_CO_3_) (700 mM). The absorbance was read at 765 nm, and the amount of TPC was estimated by using a standard curve.

### Total Sugars

The leaf powder was mixed with concentrated sulfuric acid and then neutralized by using Na_2_CO_3_ for the determination of total sugars. The contents were filtered and used to measure the optical density (OD) at 415 nm (26).

### Vitamin C

Vitamin C in the leaf powder was estimated by using the 2,6-dichlorophenolindophenol (DCPIP) method as described by Hussein et al. (27). Briefly, each molecule of vitamin C causes reduction of DCPIP into DCPIPH_2_ molecule, and this reduction was measured as a decreasing trend in absorbance at 520 nm. A standard curve of ascorbic acid (ASA) was used to measure the vitamin C concentration in the samples.

### Total Carotenoids and Lycopene

The amounts of total carotenoids and lycopene were measured by using the protocol of Nagata and Yamashita (28). The leaf powder sample (1 g) was homogenized with 10 mL of hexane–acetone mixture (6:4) and incubated at 37°C for 5 min. The contents were then filtered and their absorbance was measured at 453, 505, and 663 nm. The amounts of carotenoids and lycopene were computed by using the following equations:

$$\begin{array}{l} \text{Carotenoids}=0.216{\text{A}}_{663}-0.304{\text{A}}_{505}+\;0.452{\text{A}}_{453}\\ \text{ Lycopene}=-0.0458{\text{A}}_{663}+\;0.372{\text{A}}_{505} \end{array}$$

### Amino Acids and Minerals

Amino acid analysis was done by ion-exchange chromatography with the help of an amino acid analyzer (*Biochrom 30*+; Biochrome Ltd, Cambridge, England), and the concentration of different minerals was determined by inductively coupled plasma optical emission spectroscopy (ICP-OES; Optima 2100-DV, Perkin Elmer, Massachusetts, USA).

## Analysis of Plasma Samples

### Estimation of Non-enzymatic Antioxidants

#### Total Flavonoids

Total flavonoids were determined by adopting an AlCl3 colorimetric approach (24). The plasma samples were mixed with 100 μL of potassium acetate (1 M), 100 μL of AlCl3 (10%), and 275 μL of water. After an incubation period of 40 min at room temperature, the absorbance of the assay solution was measured at 415 nm spectrophotometrically (UV-VIS, U2800, Hitachi, Japan). The TFs were manifested as microgram rutin equivalent (RE) mL^−1^ of the sample.

#### Total Phenolic Content

Total phenolic contents were determined by using the Folin–Ciocalteu reagent method (25). Each plasma sample (100 μL) was mixed with a 10% Folin–Ciocalteu reagent (100 μL), and homogenized thoroughly by a vortex. Then samples were incubated at room temperature for 1 h after adding 800 μL Na_2_CO_3_ (700 mM), and the absorbance was measured at 765 nm. The amount of TPC was calculated as Gallic acid equivalents (GAE) by using a standard Gallic acid linear regression curve.

#### Vitamin C

Vitamin C contents were measured as ascorbic acid by following the standard protocol of Hussein et al. (27) with some necessary modifications. Ascorbic acid causes the reduction of a blue color compound DCPIP into a colorless compound DCPIPH_2_, and this transformation can be measured by the fall-off absorbance at 520 nm. Vitamin C contents were measured by using a standard calibration curve of ascorbic acid.

#### Malondialdehyde

Malondialdehyde (MDA) in blood plasma samples was assayed in terms of lipid peroxidation, which is measured through the thiobarbituric acid (TBA) reaction according to the method of Dhindsa et al. (29). For estimation of MDA, plasma samples were homogenized with TCA (0.1%) and centrifuged for 5 min at 14,462 × g. Then, 1 mL of the supernatant was mixed with TCA (20%) having 0.05% TBA. The reaction solution was heated for 30 min at 95°C and cooled quickly in an ice bath. The absorbance of the supernatant was read at 532 nm after centrifugation for 10 min at 14,462 x g, and the value of non-specific absorption at 600 nm was subtracted from it. The MDA value was calculated by using a coefficient of extinction 155 mM^−1^cm^−1^.

#### Total Antioxidant Capacity

Total antioxidant capacity (TAC) was calculated by the using 2,2-azino-bis (3-ethylbenzothiazoline-6-sulfonate) (ABTS) method as described by Dikilitas et al. (30). The assay is based on the reduction of the ABTS radical cation (ABTS^·+^) that is blue-green in color by antioxidants to its original colorless ABTS form. The assay solution composed of reagent R_1_ (mixture of glacial acetic acid and sodium acetate buffer solution), reagent R_2_ (mixture of glacial acetic acid, hydrogen peroxide, sodium phosphate buffer and ABTS), and the sample. The absorbance was read at 660 nm after mixing the contents of the tube and allowing them to stand for 6 min. The TAC was finally calculated from the standard curve of ascorbic acid and expressed as μM ascorbic acid equivalent mL^−1^.

#### Total Oxidant Status

The protocol for the estimation of total oxidant status (TOS) is based on the oxidation of iron with valence 2^+^ to an iron complex with valence 3^+^ in an acidic medium (30). Ferric ions react with xylenol orange and form a specific-colored complex. The color intensity is related to oxidant molecule quantity and can be measured spectrophotometrically. The assay mixture for determination of TOS comprised of reagent R_1_; xylenol orange solution (0.38 g in 500 μL of 25 Mm H_2_SO_4_), reagent R_2_; (o-dianisidine 0.0317 g, ferrous ammonium sulfate (II) 0.0196 g, NaCl 0.4 g, glycerol 500 μL); and the sample. The absorbance value was read at 520 nm after 5 min. The calibration curve of hydrogen peroxide (H_2_O_2_) was used to calculate TOS and is expressed in μmol H_2_O_2_ equivalent mL^−1^.

### Enzymatic Antioxidant Activity

#### Superoxide Dismutase Activity

Superoxide dismutase enzyme activity of blood plasma was measured by an inhibition assay with some modifications (31). The assay is based on the ability of SOD to inhibit the photoreduction of nitroblue tetrazolium (NBT) into formazan. Assay solution to study the inhibition of NBT consisted of 50 mM phosphate buffer (pH 7.8), 57 μM NBT, 13 mM L-methionine, riboflavin (0.004%), triton 100X (0.025%), and plasma sample (50 μL) in a total volume of 3 mL. The photoreaction was carried out in a box lined with aluminum (Al) foil and fitted with a 15-W lamp as a source of luminescence. The absorbance during photo-reduction of NBT to formazan was measured at 560 nm spectrophotometrically. One unit of SOD activity was defined as the amount of enzyme required to cause 50% inhibition of photo-chemical reduction of NBT.

#### Peroxidase Activity

Peroxidase enzyme activity in blood plasma was assayed as described by Agostini et al. (32) using guaiacol as the substrate. The reaction solution for the analysis of POD activity comprised phosphate buffer (pH 7.0, 200 mM), guaiacol (200 mM), 545 μL distilled water, H_2_O_2_ (400 mM), and 15 μL sample. The reaction was started immediately after adding the sample, and the change in absorbance of the assay mixture was read at 470 nm for 1 min after every 20 s. One unit of enzyme (POD) activity was defined as the amount of enzyme required to catalyze the oxidation of guaiacol (1 μM) at 25°C and pH 7.0 in 1 min.

#### Catalase Activity

The blood plasma sample for estimation of CAT activity was homogenized in a medium containing dithiothreitol (1 mM) and potassium phosphate buffer (pH 7.0, 50 mM). The CAT activity was assayed according to the method of Beers and Sizer (33). The assay mixture for measurement of enzyme activity was composed of H_2_O_2_ (59 mM), potassium phosphate buffer (pH 7.0, 50 mM), and 100 μl plasma sample. The change in absorbance of the assay solution was measured at 240 nm for 1 min after every 20 s, and a decrease in absorbance in 0.01 min was defined as one unit of CAT activity.

### Biochemical Parameters

#### Total Soluble Protein

Protein estimation in plasma samples was done by the dye-binding protocol of Bradford (34). For estimation of protein, 5 μL of the sample was homogenized with NaCl (0.1 N) and then 1.0 mL Bradford dye was added and mixed thoroughly. The reaction mixture was incubated at room temperature for 5 min to form a dye–protein complex and then its absorbance was read at 420 nm.

#### Total Sugars

Sugar contents of blood plasma samples were executed by the method of Folin (26) with some modifications. Briefly, the plasma sample was mixed with sulfuric acid and then neutralized with Na_2_CO_3_. Thereafter, the contents were filtered and their absorbance was measured at 415 nm.

#### Carotenoids and Lycopene

Plasma carotenoids and lycopene were calculated by using the method of Nagata and Yamashita (28). For estimation of carotenoids and lycopene, 1 mL of plasma sample was mixed with 10 mL of hexane acetone mixture (6:4) and incubated at 37°C for 5 min. The reaction mixture was then filtered and its absorbance was read at 453 nm, 505 nm, and 663 nm. The amounts of carotenoids and lycopene were computed by using the following formulae:

$$\begin{array}{l} \text{Carotenoids}=0.216{\text{A}}_{663}-0.304{\text{A}}_{505}+\;0.452{\text{A}}_{453}\\ \text{ Lycopene}=-0.0458{\text{A}}_{663}+\;0.372{\text{A}}_{505} \end{array}$$

#### Progesterone Profile

The progesterone concentration of plasma samples was measured by radioimmunoassay (Video gamma/Rack, I'acn, Italy) using commercially available kits (DIAsource, S.A, Belgium). The sensitivity of immunoassay was 0.05 ng mL^−1^, and the intra-assay and inter-assay variation coefficients were 6.5 and 8.2%, respectively. The cross-reactivity with other steroid hormones was <0.01–15%.

### Hydrolytic Enzymes

#### Protease

The protease enzyme activity was estimated by using a casein substrate (35). The assay is based on the digestion of casein by protease enzyme and the release of an amino acid “tyrosine” that reacts with Folin's reagent to produce a blue-colored product which is measured and quantified at 660 nm. A calibration curve of tyrosine was drawn by using its standard solutions to measure the protease activity from blood plasma samples.

#### Esterase

The esterase (α & β) activity was determined by using α-naphthyl acetate and β-naphthyl acetate as substrates (36). The reaction solution comprised of enzyme extract (plasma) and substrate (1% acetone, 30 mM naphthyl acetate (α & β), and 0.04 M potassium phosphate buffer) was incubated at room temperature for 15 min in the dark. Then, 1 mL of staining solution [sodium dodecyl sulfate (5%) and fast blue BB (1%) in the ratio of 5:2] was added in both the reaction mixture and the blank control (substrate solution and phosphate buffer) and incubated again in the dark for 15 min at room temperature. The absorbance of the reaction mixture was read at 590 nm, and the enzyme activity was calculated in μM/min per mL of plasma by using a standard curve.

#### Amylase

Plasma amylase activity was assessed by using tris-malate buffer (0.2 M, pH 7.2), which served as an assay cum extraction medium (37). For the measurement of amylase activity, 1 mL of plasma was mixed with 1 mL of substrate solution (0.15% starch) and incubated for 10 min at 37°C. Then, OD was read at 620 nm after adding quenching reagent. The activity of the enzyme was expressed in milligrams of starch degraded at min^−1^per mL of blood plasma.

### Statistical Analysis

The statistical analyses were performed with SPSS version 20. All the experimental procedures were performed in triplicates, and data were analyzed by one way-ANOVA with repeated measures to access the differences among various treatments on specific days. The results are presented in the tables as means ± SE, and the treatments with *p* < 0.05 were regarded statistically significant.

## Results

### Non-enzymatic Antioxidants

The impact of MOLP supplementation on plasma non-enzymatic antioxidant indices is shown in Table 4. The MOLP significantly increased plasma TF in the 1.6% MOLP supplemented group as well as in the 3.2% MOLP supplemented group in comparison with the control group on day 40 and day 60 of the experiment (*p* < 0.05). The highest value of TF was observed on day 60 in the 3.2% MOLP supplemented group. The TPC was significantly high throughout the experiment in the 3.2% MOLP supplemented group (*p* < 0.05), and the impact of 1.6% supplementation remained non-significant as compared with the control group (*p* > 0.05). Plasma vitamin C level increased significantly with 3.2% supplementation from the day of conception (day 0), whereas treatment with 1.6% MOLP exhibited a significant impact from day 20 to 60 of the gestation (*p* < 0.05). The MDA concentration declined significantly after MOLP supplementation from day 20 of the gestation in response to the control group (*p* < 0.05). Plasma TAC increased significantly during the experiment in supplemented groups as compared with the control group (*p* < 0.05) till it reached its peak on day 60 in the 3.2% MOLP supplemented group. The TOS value decreased significantly with the advancement in pregnancy in supplemented groups from day 20 till the end of the experiment on day 60 (*p* < 0.05).

**Table 4:** Plasma non-enzymatic antioxidant indices of pregnant beetal goats.

**Non-enzymatic antioxidants**	**Levels of** ***Moringa oliefera*** **leaf powder supplementation**			**SEM**	***P*-value**
	**0%**	**1.6%**	**3.2%**		
**Day 0**					
Total flavonoids (μg/mL)	241.89 ± 0.75	245.02 ± 0.74	247.01 ± 0.14	0.43	0.061
Total phenolic contents (μM/mL)	5872 ± 0.82^b^	5881.25 ± 1.65^b^	5894 ± 1.41^a^	0.86	0.016
Vitamin C (μg/mL)	827.25 ± 0.75^b^	830 ± 0.41^b^	839.75 ± 0.75^a^	0.49	0.006
Malondialdehyde (μM/mL)	5.50 ± 0.09	5.37 ± 0.19	5.30 ± 0.10	0.09	0.201
Total antioxidant capacity (μM/mL)	1.31 ± 0.01^c^	1.42 ± 0.03^b^	1.55 ± 0.02^a^	0.01	0.002
Total oxidant status (μM/mL)	1, 457 ± 0.91	1, 455.25 ± 0.85	1, 450.75 ± 2.21	1.03	0.226
**Day 20**					
Total flavonoids (μg/mL)	260.65 ± 0.87	273.57 ± 3.92	281.45 ± 6.91	2.26	0.096
Total phenolic contents (μM/mL)	5, 901 ± 0.91^b^	5, 909.5 ± 2.21^b^	5, 924.75 ± 0.63^a^	0.58	0.004
Vitamin C (μg/mL)	825.5 ± 0.96^c^	841 ± 0.91^b^	853.75 ± 0.75^a^	0.25	0.001
Malondialdehyde (μM/mL)	7.31 ± 0.47^b^	5.08 ± 0.45^a^	4.51 ± 0.54^a^	0.45	0.048
Total antioxidant capacity (μM/mL)	1.15 ± 0.02^c^	1.57 ± 0.03^b^	1.88 ± 0.03^a^	0.01	0.001
Total oxidant status (μM/mL)	1475 ± 0.81^c^	1435.5 ± 0.28^b^	1419.5 ± 0.64^a^	0.13	0.002
**Day 40**					
Total flavonoids (μg/mL)	275.86 ± 0.99^c^	283.39 ± 0.70^b^	304.64 ± 1.63^a^	0.54	0.004
Total phenolic contents (μM/mL)	5, 916 ± 0.82^b^	5, 918 ± 1.35^b^	5, 935.75 ± 0.48^a^	0.53	0.006
Vitamin C (μg/mL)	822 ± 0.41^c^	848.75 ± 0.85^b^	865 ± 0.71^a^	0.34	0.001
Malondialdehyde (μM/mL)	8.03 ± 0.27^c^	4.65 ± 0.40^b^	3.21 ± 0.21^a^	0.28	<0.001
Total antioxidant capacity (μM/mL)	1.21 ± 0.04^c^	1.70 ± 0.03^b^	2.07 ± 0.02^a^	0.03	0.001
Total oxidant status (μM/mL)	1, 487.5 ± 0.64^c^	1, 418.75 ± 0.75^b^	1, 395 ± 0.91^a^	0.58	<0.001
**Day 60**					
Total flavonoids (μg/mL)	281.11 ± 0.67^c^	296.51 ± 0.89^b^	311.99 ± 0.51^a^	0.54	0.002
Total phenolic contents (μM/mL)	5, 914.5 ± 1.55^b^	5, 922 ± 2.67^b^	5, 948 ± 0.41^a^	1.19	0.009
Vitamin C (μg/mL)	818.5 ± 0.64^c^	850.25 ± 0.48^b^	871.75 ± 0.47^a^	0.39	<0.001
Malondialdehyde (μM/mL)	8.34 ± 0.64^c^	4.31 ± 0.21^b^	1.95 ± 0.41^a^	0.31	0.038
Total antioxidant capacity (μM/mL)	1.19 ± 0.07^c^	1.72 ± 0.04^b^	2.29 ± 0.05^a^	0.03	0.004
Total oxidant status (μM/mL)	1, 505.5 ± 0.86^c^	1, 407 ± 0.40^b^	1, 376.25 ± 0.47^a^	0.43	<0.001

*Means with different superscript letters (a, b, c) within the same row differ significantly at P ≤ 0.05*.

### Enzymatic Antioxidants

Enzymatic antioxidant activities in the blood plasma of control and supplemented groups are shown in Table 5. Plasma SOD activity increased significantly in supplemented groups as compared with the control group (*p* < 0.05) throughout the experiment; however the increase was nonsignificant within supplemented groups on the day of conception (day 0) and on day 20 of gestation (*p* > 0.05). The increase in plasma POD activity remained nonsignificant between both (1.6 and 3.2%) treatments (*p* > 0.05) but remained significant in comparison with the control group during the entire experiment (*p* < 0.05). The MOLP supplementation did not exhibit any significant impact on CAT activity till day 20 (*p* > 0.05). After that, 3.2% treatment showed a significant effect on day 40 and day 60 in response to the control group (*p* < 0.05).

**Table 5:** Plasma enzymatic antioxidant indices of pregnant beetal goats.

**Enzymatic antioxidants (Units/mL)**	**Levels of** ***Moringa oleifera*** **leaf powder supplementation**			**SEM**	***p*-value**
	**0%**	**1.6%**	**3.2%**		
**Day 0**					
SOD	20.18 ± 0.28^b^	22.89 ± 0.36^a^	24.01 ± 0.67^a^	0.34	0.040
POD	216.79 ± 1.01^b^	226.54 ± 0.84^a^	232.12 ± 1.83^a^	0.62	0.012
CAT	35.50 ± 0.64	32 ± 3.02	35.25 ± 0.48	1.19	0.562
**Day 20**					
SOD	19.06 ± 0.39^b^	22.81 ± 0.35^a^	27.73 ± 1.46^a^	0.48	0.018
POD	232.5 ± 0.87^b^	262.43 ± 1.19^a^	267.03 ± 0.62^a^	0.67	0.001
CAT	37 ± 0.91	36.25 ± 0.85	41 ± 0.70	0.21	0.205
**Day 40**					
SOD	17.91 ± 0.20^c^	26.81 ± 0.33^b^	31.83 ± 0.32^a^	0.23	0.002
POD	240.46 ± 0.98^b^	274.65 ± 0.76^a^	281.59 ± 2.02^a^	1.04	0.003
CAT	37 ± 0.71^b^	39 ± 0.40^b^	46 ± 0.41^a^	0.19	0.029
**Day 60**					
SOD	14.93 ± 0.19^c^	28.68 ± 0.35^b^	34.80 ± 0.42^a^	0.15	0.002
POD	246.51 ± 0.56^b^	283.12 ± 0.93^a^	290.48 ± 1.88^a^	0.81	0.001
CAT	40.50 ± 0.87^b^	42.50 ± 0.64^b^	51 ± 0.41^a^	0.24	0.019

*Means with different superscript letters (a, b, c) within the same row differ significantly at P ≤ 0.05*.

*SOD, Superoxide dismutase; POD, Peroxidase; CAT, Catalase*.

### Biochemical Parameters

The plasma biochemical profile of different parameters observed during the experiment is depicted in Table 6. The supplementation of MOLP significantly increased plasma protein contents from day 40 to day 60 of gestation in the 1.6% and 3.2% MOLP supplemented groups when compared with the control group (*p* < 0.05). A significant increase in plasma total sugars and carotenoids was observed throughout the experiment with 3.2% supplementation (*p* < 0.05), whereas 1.6% supplementation showed significant results on day 60 (*p* < 0.05). The amount of lycopene in plasma elevated significantly after feeding MOLP from day 20 in the 3.2% MOLP supplemented group and from day 40 in the 1.6% MOLP supplemented group (*p* < 0.05).

**Table 6:** Plasma biochemical indices of pregnant beetal goats.

**Biochemicals**	**Levels of** ***Moringa oleifera*** **leaf powder supplementation**			**SEM**	***p*-value**
	**0%**	**1.6%**	**3.2%**		
**Day 0**					
Total soluble proteins (mg/mL)	59.40 ± 0.37	61.63 ± 0.46	62.13 ± 0.99	0.21	0.071
Total sugars (mg/mL)	4.45 ± 0.08^b^	4.60 ± 0.09^b^	5.18 ± 0.06^a^	0.04	0.038
Carotenoids (μg/mL)	158.10 ± 0.59^b^	160.11 ± 0.51^b^	165.69 ± 0.52^a^	0.15	0.040
Lycopene (μg/mL)	100 ± 0.41	103 ± 0.82	110 ± 1.78	0.39	0.064
**Day 20**					
Total soluble proteins (mg/mL)	60.48 ± 0.34	61.80 ± 0.18	63.13 ± 0.45	0.15	0.089
Total sugars (mg/mL)	4.11 ± 0.41^b^	5.57 ± 0.12^b^	6.47 ± 0.02^a^	0.15	0.043
Carotenoids (μg/mL)	156.53 ± 1.38^b^	163.79 ± 0.45^b^	177.49 ± 0.71^a^	0.21	0.007
Lycopene (μg/mL)	98 ± 1.15^b^	105.25 ± 1.79^b^	128.75 ± 1.48^a^	0.91	0.004
**Day 40**					
Total soluble proteins (mg/mL)	56.25 ± 0.96^c^	64.67 ± 0.41^b^	68.03 ± 0.79^a^	0.63	0.013
Total sugars (mg/mL)	4.01 ± 0.29^b^	5.62 ± 0.22^b^	6.95 ± 0.09^a^	0.07	0.013
Carotenoids (μg/mL)	160.94 ± 0.44^b^	169.9 ± 1.89^b^	188.81 ± 0.63^a^	1.24	0.017
Lycopene (μg/mL)	95.5 ± 0.64^c^	118.75 ± 0.75^b^	132.25 ± 0.63^a^	0.29	<0.001
**Day 60**					
Total soluble proteins (mg/mL)	51.48 ± 0.74^c^	66.31 ± 0.29^b^	71.80 ± 0.64^a^	0.26	0.006
Total sugars (mg/mL)	3.95 ± 0.22^c^	5.74 ± 0.08^b^	7.09 ± 0.24^a^	0.09	0.019
Carotenoids (μg/mL)	166.73 ± 0.48^c^	182.45 ± 0.55^b^	191.01 ± 0.85^a^	0.54	0.002
Lycopene (μg/mL)	97.75 ± 0.48^c^	127 ± 0.91^b^	145.75 ± 0.48^a^	0.44	<0.001

*Means with different superscript letters (a, b, c) within the same row differ significantly at P ≤ 0.05*.

The beneficial effect of MOLP supplementation on plasma progesterone concentration in early pregnant goats is displayed in Table 7. The exposure of the animals with 3.2% supplementation showed significant effect and more effectively enhanced plasma progesterone level throughout the experiment than that of the animals with the 1.6% MOLP supplementation and the control group (*p* < 0.05).

**Table 7:** Plasma progesterone (ng/mL) profile of pregnant beetal goats.

**Days**	**Levels of** ***Moringa oleifera*** **leaf powder supplementation**			**SEM**	***p*-value**
	**0%**	**1.6%**	**3.2%**		
0	0.65 ± 0.02^b^	0.69 ± 0.04^b^	0.78 ± 0.03^a^	0.03	0.025
15	1.84 ± 0.09^b^	2.08 ± 0.04^b^	2.44 ± 0.03^a^	0.05	0.023
30	3.31 ± 0.05^b^	3.78 ± 0.13^b^	4.55 ± 0.04^a^	0.05	0.006
45	5.45 ± 0.04^b^	5.72 ± 0.07^b^	6.41 ± 0.13^a^	0.06	0.022

*Means with different superscript letters (a, b) within the same row differ significantly at P ≤ 0.05*.

### Hydrolytic Enzymes

The activities of hydrolytic enzymes of both supplemented and control groups are illustrated in Table 8. The protease activity was significantly higher in the 3.2% MOLP supplemented group from day 20 till the completion of the experiment on day 60 of gestation as compared with the control group (*p* < 0.05). However, MOLP supplementation did not show any significant impact on esterase activity across the experiment in both the supplemented groups (*p* > 0.05). The amylase activity was not influenced by 1.6% supplementation when compared with the control group (*p* > 0.05), whereas 3.2% supplementation slightly increased the amylase activity and produced a significant impact on day 60 of the experiment (*p* < 0.05).

**Table 8:** Plasma hydrolytic enzyme activities of pregnant beetal goats.

**Enzymes**	**Levels of** ***Moringa oleifera*** **leaf powder supplementation**			**SEM**	***p*-value**
	**0%**	**1.6%**	**3.2%**		
**Day 0**					
Protease (U/mL)	235.25 ± 1.65	239.75 ± 0.48	240.25 ± 0.85	0.66	0.206
Esterase (μM/min/mL)	678 ± 0.91	681 ± 1.58	675.25 ± 0.63	0.79	0.135
Amylase (mg/min/mL)	1.26 ± 0.02	1.24 ± 0.01	1.28 ± 0.02	0.01	0.102
**Day 20**					
Protease (U/mL)	235 ± 2.12^b^	240.75 ± 0.75^b^	251 ± 0.71^a^	0.89	0.022
Esterase (μM/min/mL)	675 ± 2.67	677.75 ± 0.85	679.25 ± 0.85	1.01	0.549
Amylase (mg/min/mL)	1.28 ± 0.02	1.28 ± 0.03	1.29 ± 0.01	0.02	0.781
**Day 40**					
Protease (U/mL)	237.25 ± 0.75^b^	242.75 ± 1.31^b^	253.75 ± 0.75^a^	0.55	0.005
Esterase (μM/min/mL)	679.5 ± 0.86	674.75 ± 0.75	673.75 ± 0.63	0.30	0.069
Amylase (mg/min/mL)	1.28 ± 0.03	1.32 ± 0.01	1.32 ± 0.01	0.02	0.494
**Day 60**					
Protease (U/mL)	240.75 ± 0.85^b^	242.75 ± 0.95^b^	256.5 ± 0.64^a^	0.68	0.006
Esterase (μM/min/mL)	671.25 ± 0.58	669.75 ± 0.25	667 ± 0.48	0.24	0.152
Amylase (mg/min/mL)	1.3 ± 0.01^b^	1.32 ± 0.02^b^	1.44 ± 0.02^a^	0.01	0.037

*Means with different superscript letters (a, b) within the same row differ significantly at P ≤ 0.05*.

## Discussion

Animals are more vulnerable to oxidative stress during pregnancy, especially in early gestational stages due to certain physiological changes in the maternal body. The quality of feedstuff plays an important role during this critical stage of pregnancy (38). The MO leaves possess several nutritional and medicinal properties that have been ascribed to the presence of different phytochemicals (39, 40). It is evident from the results of this study that supplementation of the diet with MOLP improved the plasma non-enzymatic antioxidants status during early gestation in goats. The elevated level of plasma flavonoids and phenolics enhanced the survival and growth rate of developing embryos by suppressing the immense production of nitric oxide (NO) and hydroxyl (OH) radicals as a result of early pregnancy stress (41). The increase in plasma flavonoids and phenolic contents in response to MOLP supplementation was in accordance with the findings of another study, in which MO leaf extract modified the antioxidant capacity in rats by improving the plasma flavonoids and phenolic contents under different oxidative stress conditions (42).

The plasma vitamin C level drops promptly with the advancement in pregnancy, which makes the mother as well as the developing fetus more prone to detrimental effects of oxidative stress (43). However, a significant improvement in plasma vitamin C contents in the supplemented groups demonstrated that MOLP, being a rich source of vitamin C, has the potential to provide protection from the harmful effects of oxidative stress during pregnancy. Our results were in agreement with the findings of Babiker et al. (44), who reported the positive impacts of MO supplementation on plasma and milk vitamin C contents in ewes.

Furthermore, MOLP supplementation also improved plasma TAC by limiting the production of MDA, a product of lipid peroxidation (LPO). MDA produced during pregnancy is more stable and highly reactive for biomolecules (proteins or DNA); thus, it leads to the development of certain pathological conditions that finally cause abortion (45, 46). The results of this study evidenced that MOLP supplementation also prevented the elevation of plasma MDA to promote the activities of enzymatic (SOD, POD, and CAT) antioxidants. Both the enzymatic and non-enzymatic antioxidant defense systems work side by side in attenuating oxidative stress to make conditions more favorable for the growth of the developing fetus (47). The SOD enzyme during the process of embryo implantation neutralizes ROS by stimulating the transformation of superoxide anion to hydrogen peroxide (H_2_O_2_), which is further metabolized into water and oxygen by POD and CAT (48). The successful implantation of an embryo on the endometrium of the uterus is dependent on SOD, POD, and CAT activities (49). The improvement in the enzymatic antioxidant defense system was also observed in rabbits, dairy cows, and sows following Moringa leaf supplementation in different proportions (18, 50, 51).

The excessive production of free radicals (ROS and RNS) during early gestation as a result of physiological changes increased the plasma TOS (52, 53). The high level of TOS is an indicator of oxidative stress. The results of this study depicted that MOLP supplementation decreased TOS values in the supplemented groups by restricting the production of ROS and RNS, as reported in some previous studies performed in ruminants (54, 55).

Blood biochemical analysis is a definite method for predicting the health status of animals (56). The MO supplementation has a significant impact on blood biochemistry (57). The increase in plasma protein level after MOLP supplementation indicated that MOLP was a lavish source of proteins, and its supplementation increased the plasma protein level by decreasing blood urea nitrogen (BUN) contents (50). The BUN is considered as an indicator of the balance between amino acid metabolism and protein synthesis (58). Pregnancy is considered to be a period of severe metabolic and protein alterations in the body (59). The decrease in plasma protein level with the advancement of gestation in the control group was due to the high demand for amino acids, which were transferred from the mother to the developing fetus for the synthesis of numerous proteins required for the proper growth of the fetus and for the maintenance of a healthy pregnancy (60). The high protein profile of MOLP makes it an ideal substitute for rapeseed and soybean meals for ruminants.

The significant increase in plasma sugar contents by dietary supplementation with MOLP specified its positive effects in improving the energy status of the body. During gestation, there is a rapid increase in metabolic rate to fulfill the high energy demands of pregnancy. However, if the provision of feed is not according to the requirements, the mobilization of body fat starts, which results in the production of non-esterified fatty acids (NEFAs). NEFAs are responsible for the development of ketosis and some other metabolic diseases that may adversely affect the pregnancy and future health of the offspring (61, 62). The provision of MOLP met the energy requirements more efficiently in early pregnancy by improving the plasma sugar contents. Our findings were in line with the results of a previous study on Jersey cows (63). In addition, MOLP supplementation also improved the bioavailability of carotenoids and lycopene to ameliorate metabolic and hematological perturbations by reducing LPO and inflammatory cytokines (64).

The progesterone hormone plays a key role in maintaining pregnancy (65). The ROS produced during early gestation prevents the implantation of the embryo by suppressing the production of progesterone from the corpus luteum (66). The high level of plasma progesterone concentration in treatment groups indicates a strong correlation with MOLP supplementation during early pregnancy. In this experiment, a 100% pregnancy rate was observed in the 3.2% MOLP supplemented group, while 80 and 70% pregnancy rates were noticed in the 1.6% M OLP supplemented and control groups, respectively. The results of our experiment are in accordance with the findings in rabbits supplemented with Moringa leaf. An increase in conception rate along with plasma progesterone profile was observed (67).

The protease enzymes from MO leaves help in maintaining body hemostasis during gestational stress by acting as an important component of the complement system. These enzymes also help in hydrolyzing and removing apoptotic products of oxidative stress from the body (68, 69). The high protease activity in supplemented groups indicate the beneficial impact of MOLP in maintaining body conditions by modifying and stabilizing plasma protease activity during early gestation in pregnant goats. Amylase enzyme maintains blood glucose level according to the energy requirements of the body by converting carbohydrates into glucose. The results of an *in-vitro* study indicated that MO leaf extract inhibited amylase activity at a concentration of 220 μg/mL of blood plasma, and below this level, it potentiated the activity of the enzyme to a certain extent (70). The MOLP used within the limit to increase the amylase activity denotes its supportive role in potentiating enzyme activity.

## Conclusion

The supplementation of the basal diet with 3.2% MOLP improved the antioxidant defense system and blood biochemical indices during early gestation in Beetal goats. In addition, it also enhanced the progesterone profile by suppressing the production of reactive oxygen species. These findings suggest that MOLP is a promising agent to improve the reproductive performance of goats during early pregnancy. However, further studies are required on a large flock of animals with different concentrations of MOLP to dig out the molecular aspects of attenuating early pregnancy stress and improving fertility.

## Data Availability Statement

The raw data supporting the conclusions of this article will be made available by the authors, without undue reservation.

## Author Contributions

AA: conduct experiment, data aggregation, statistical analysis, and wrote manuscript. AH: lab analysis. TH: design experiment, monitoring experiment, and revised manuscript. All authors contributed to the article and approved the submitted version.

## Conflict of Interest

The authors declare that the research was conducted in the absence of any commercial or financial relationships that could be construed as a potential conflict of interest.

## Publisher's Note

All claims expressed in this article are solely those of the authors and do not necessarily represent those of their affiliated organizations, or those of the publisher, the editors and the reviewers. Any product that may be evaluated in this article, or claim that may be made by its manufacturer, is not guaranteed or endorsed by the publisher.
